# Response to the Letter to the Editor by Prof. Dr. Aydin Gülses regarding our article ‘The effect of age on third molar extraction difficulty: A retrospective cross-sectional cohort study’

**DOI:** 10.2340/aos.v85.45935

**Published:** 2026-04-29

**Authors:** Abiel Noro, Johanna Snäll, Irja Ventä

**Affiliations:** a Department of Oral and Maxillofacial Diseases, Faculty of Medicine, University of Helsinki, Helsinki, Finland; b Department of Oral and Maxillofacial Diseases, University of Helsinki and Helsinki University Hospital, Helsinki, Finland

**Keywords:** Molar, third, tooth extraction, primary health care, age group, surgery, oral

We thank Prof. Dr. Aydin Gülses for his interest in our recent publication and for his thoughtful comments [1]. It is a pleasure to note that our publication has stimulated discussion.

We agree that the points raised regarding bone quality, systemic health, and medication-related risks are highly relevant and deserve careful consideration in clinical practice. However, it should be emphasized that our study was retrospective in nature and based on register data and, therefore, did not allow for detailed assessment of individual patient factors such as bone mineral density, systemic conditions, or medication use. We acknowledge that bone mineral density, among other factors, influences surgical difficulty.

The primary aim of our study was to evaluate the perceived difficulty of third molar extractions across age groups. For this purpose, we used treatment code data, enabling analysis of a large number of extractions. As noted in our results, simple mandibular third molar extractions were associated with reduced technical difficulty in older patients. One possible explanation for this finding may be age-related periodontal changes, such as periodontitis, which could facilitate tooth removal in some cases [2]. Importantly, our findings should not be interpreted as suggesting that extractions in older patients are generally easier or safer. As discussed in our study, several factors, including reduced periodontal ligament quality, decreased bone elasticity, hypercementosis, and ankylosis, are known to increase the difficulty of extractions in older patients [3–7]. Furthermore, we noted in the introduction that increasing age is associated with a higher risk of complications [2, 8–10].

It should also be stressed that the aim of our study was not to provide recommendations for or against third molar removal, but rather to contribute to the limited body of literature on extraction difficulty, including both simple and surgical procedures in primary care. Finally, we fully agree that third molar extraction should always be based on clear clinical indications and approached with caution. Prophylactic extraction in elderly individuals is generally not recommended, and comprehensive patient evaluation remains essential.

In conclusion, we agree that careful, individualized assessment of each patient is crucial when planning third molar extractions. The opportunity to clarify the scope and interpretation of these findings is appreciated.
